# A high-throughput assay for directly monitoring nucleolar rRNA biogenesis

**DOI:** 10.1098/rsob.210305

**Published:** 2022-01-26

**Authors:** Carson J. Bryant, Mason A. McCool, Laura Abriola, Yulia V. Surovtseva, Susan J. Baserga

**Affiliations:** ^1^ Department of Molecular Biophysics and Biochemistry, Yale School of Medicine, 333 Cedar Street, New Haven, CT, USA; ^2^ Yale Center for Molecular Discovery, Yale University, West Haven, CT, USA; ^3^ Department of Genetics, Yale School of Medicine, New Haven, CT, USA; ^4^ Department of Therapeutic Radiology, Yale School of Medicine, New Haven, CT, USA

**Keywords:** ribosome biogenesis, nucleolus, pre-rRNA transcription, pre-rRNA processing, RNA polymerase 1, high-throughput screening

## Abstract

Studies of the regulation of nucleolar function are critical for ascertaining clearer insights into the basic biological underpinnings of ribosome biogenesis (RB), and for future development of therapeutics to treat cancer and ribosomopathies. A number of high-throughput primary assays based on morphological alterations of the nucleolus can indirectly identify hits affecting RB. However, there is a need for a more direct high-throughput assay for a nucleolar function to further evaluate hits. Previous reports have monitored nucleolar rRNA biogenesis using 5-ethynyl uridine (5-EU) in low-throughput. We report a miniaturized, high-throughput 5-EU assay that enables specific calculation of nucleolar rRNA biogenesis inhibition, based on co-staining of the nucleolar protein fibrillarin (FBL). The assay uses two siRNA controls: a negative non-targeting siRNA control and a positive siRNA control targeting RNA Polymerase 1 (RNAP1; *POLR1A*), and specifically quantifies median 5-EU signal within nucleoli. Maximum nuclear 5-EU signal can also be used to monitor the effects of putative small-molecule inhibitors of RNAP1, like BMH-21, or other treatment conditions that cause FBL dispersion. We validate the 5-EU assay on 68 predominately nucleolar hits from a high-throughput primary screen, showing that 58/68 hits significantly inhibit nucleolar rRNA biogenesis. Our new method establishes direct quantification of nucleolar function in high-throughput, facilitating closer study of RB in health and disease.

## Graphical abstract

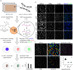

## 1. Introduction

Cells of all organisms manufacture mature ribosomes, the core machinery of protein translation, through a process known as ribosome biogenesis (RB) (reviewed in [1,2]). In eukaryotic cells, the first steps of RB occur in the nucleolus, a membraneless nuclear organelle discovered in the 1830s (reviewed in [3–5]), where RNA Polymerase 1 (RNAP1) transcribes the primary pre-ribosomal RNA (pre-rRNA) precursor (reviewed in [6–8]). Subsequently, a series of RNA processing and modification steps transpire, largely within the nucleolus, to create the mature cytoplasmic 18S, 5.8S, and 28S rRNA molecules in human cells [9,10]. Ribosomal proteins (RPs) bind (pre-)rRNA substrates in a hierarchical progression throughout this maturation process, bolstering the stability of the nascent transcript by chaperoning its folding away from incorrect energetically minimized conformations [11,12]. Dysregulation of RB, and particularly of RNAP1 transcription, is a causative factor in a myriad of human disease states, including cancer [13–18], ageing [7,19] and rare diseases called ribosomopathies [20–22].

Given the importance of nucleolar function in human health and disease, the creation of more robust tools for measuring rRNA biogenesis within the nucleolus is essential for understanding the basic biological mechanisms through which RB can be regulated, as well as for developing next-generation small-molecule or biologic therapeutics. In the past decade, a cadre of studies using high-throughput screening (HTS) has elucidated novel mechanisms through which human RB is regulated [23–26]; several candidate therapeutics targeting the nucleolus have also been discovered with HTS chemical library or natural product campaigns [27–31]. While several HTS modalities for monitoring nucleolar form and morphology have been described [25,32,33], none of these platforms directly measure nucleolar rRNA biogenesis, or the synthesis and accumulation of nascent pre-rRNA within the nucleolus. To date, the lack of a direct high-throughput assay for nucleolar rRNA biogenesis constrains researchers' ability to select for and validate the most promising candidate regulators of RB.

To monitor nucleolar function in a high-throughput manner, we sought to adapt a 5-ethynyl uridine (5-EU) assay for nucleolar rRNA biogenesis to an accessible, miniaturized format. The 5-EU assay has been successfully used to quantify changes in nucleolar transcriptional activity by several other groups in a variety of systems including human tissue culture cells [32,34–41], primary neurons [42], porcine fetal fibroblasts [43], *Drosophila melanogaster* ovarian stem cells [44], and plant seedlings [45,46]. A key limitation in almost all of these studies is that total cellular or total nuclear 5-EU is quantified, rather than solely nucleolar 5-EU. Because only nucleolar signal corresponds to biogenesis of the primary pre-rRNA, quantifying total 5-EU leads to an increased background from nascent transcription by RNAPs besides RNAP1. Additionally, the computational methods used for image segmentation and quantification have varied widely and include custom MATLAB scripts, manual definition of regions-of-interest in ImageJ, and image multiplication in Adobe Photoshop, further limiting assay accessibility and reproducibility across research groups.

To improve upon these limitations, we present a miniaturized, high-throughput-ready 5-EU assay that selectively measures nucleolar rRNA biogenesis by co-staining for the nucleolar protein fibrillarin (FBL). In addition, we provide an analysis pipeline for the open-source image analysis software CellProfiler [47] that provides a facile and reproducible framework for quantifying nucleolar 5-EU levels. We validate our assay by depleting 68 known RB factors including core RNAP1 machinery, assembly factors, and RPs, demonstrating robust and reproducible results for specifically measuring nucleolar rRNA biogenesis. Strikingly, we find that nucleolar 5-EU incorporation is sensitive to defects not only in RNAP1 transcription (producing strong percentage inhibition), but also to aberrant pre-rRNA processing and ribosome assembly (producing milder percentage inhibition). We underscore that changes in pre-rRNA synthesis or in pre-rRNA stability can affect nucleolar pre-rRNA accumulation, and therefore nucleolar rRNA biogenesis is sensitive to alterations in fundamental RB subprocesses. Our results prompt an expansion of the field's conceptualization of nucleolar 5-EU incorporation experiments in general, which, at measurable time points, report not only on RNAP1 transcription, but more broadly on nucleolar rRNA biogenesis. Overall, our miniaturized 5-EU assay expands the dimensionality of HTS experiments studying the nucleolus and will accelerate the discovery of novel RB regulators and targeted therapeutics.

## 2. Results

### 2.1. A high-content assay to quantify nucleolar rRNA biogenesis

**Figure 1.**
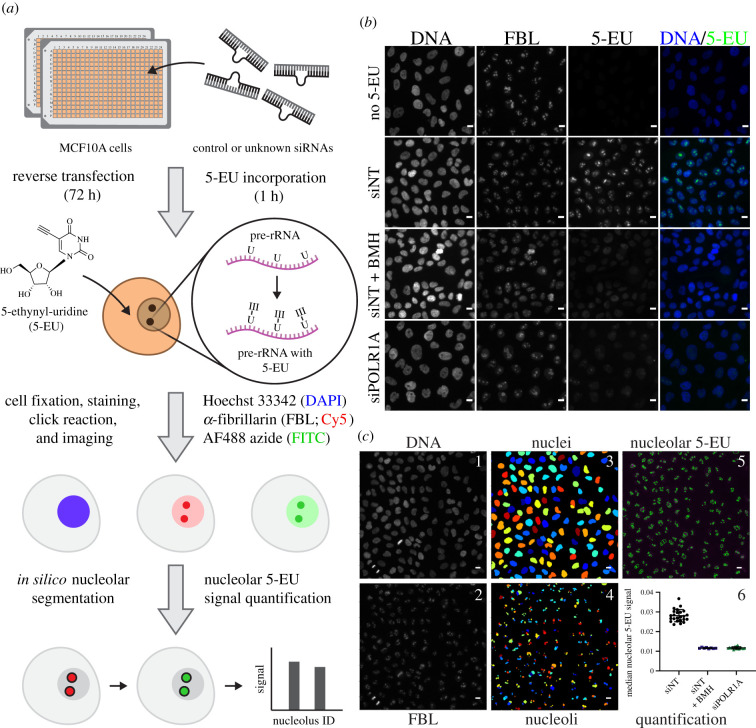
A high-throughput assay for nucleolar rRNA biogenesis using 5-ethynyl uridine (5-EU). (*a*) Schematic of the 5-EU assay protocol. MCF10A cells are reverse-transfected in 384-well imaging plates with control or unknown siRNAs for 72 h. Following target depletion, 5-EU is incorporated into nascent RNA transcripts for 1 h, with the majority of label incorporated into nascent pre-ribosomal RNA (pre-rRNA). Treated cells are fixed and stained for DNA (Hoechst 33342, DAPI channel) and the nucleolar protein FBL (Cy5 channel). 5-EU in nascent transcripts is conjugated to an azide fluorophore (AF488 azide, FITC channel) via a copper-catalysed click reaction. After fluorescent imaging, cell nuclei and nucleoli are segmented *in silico* with CellProfiler, and nucleolar-specific 5-EU signal is quantified for each nucleolus object identified. (*b*) RNAP1 inhibition specifically inhibits nucleolar 5-EU incorporation. No 5-EU, experiment without 1 h 5-EU incorporation. Treatment with a non-targeting siRNA (siNT) leads to a high 5-EU signal within the nucleolus and moderate nucleoplasmic background signal. Acute treatment with BMH-21 (siNT + BMH) or siRNA-mediated depletion of POLR1A (siPOLR1A) decreases nucleolar 5-EU signal, although nucleoplasmic background remains. DNA (Hoechst staining), FBL (staining), 5-EU (5-EU staining) and DNA/5-EU (combined Hoechst and EU staining). Scale bars, 10 µm. (*c*) Schematic of CellProfiler segmentation and nucleolar 5-EU quantification. Panels 1 and 2, raw images of DNA and FBL staining. Panels 3 and 4, nuclei or nucleoli segmented by CellProfiler from DNA or FBL staining, respectively. Rainbow colouring identifies object number. Panel 5, overlay of segmented nucleoli (green) on top of 5-EU staining (magenta). Panel 6, quantification of median nucleolar 5-EU signal for nucleoli in cells treated with siNT, siNT and BMH-21, or siPOLR1A. *n* = 24, 8 or 16 wells, respectively. Scale bars, 10 µm.

In order to achieve specific quantification of nucleolar rRNA biogenesis, we introduced a 5-EU labelling step into our previously established screening platform for counting nucleolar number [25], which uses CellProfiler [47] to segment nuclei and nucleoli in images of cells immunofluorescently stained for DNA and the nucleolar protein FBL (figure 1*a*). In our new protocol, MCF10A breast epithelial cells are reverse-transfected with siRNA duplexes for 72 h. For 1 h following the transfection period, the cells are treated with 1 mM 5-EU, which is incorporated into nascent transcripts. Since the bulk of cellular transcription occurs in the nucleolus, most of the 5-EU label is incorporated into nucleolar nascent pre-rRNA (figure 1*a*). The cells are fixed and immunofluorescently stained for DNA and FBL, after which nascent RNA is visualized *in situ* by performing a bio-orthogonal click reaction to covalently label the 5-EU alkyne moiety with an azide fluorophore (AF488 azide) (figure 1*a*). The cells are then imaged and analysed with CellProfiler to specifically quantify nucleolar rRNA biogenesis across all control and unknown wells. CellProfiler is known for its ease-of-use and modular adaptability [48,49], making it suitable for inclusion in a standardized, broadly accessible protocol.

We optimized our 5-EU assay to use a non-targeting siRNA as a negative control (siNT), and a siRNA targeting *POLR1A*, the largest subunit of RNAP1 also known as RPA194, as a positive control (siPOLR1A) (figure 1*b*). RNAP1 inhibition by POLR1A depletion strongly reduces the nucleolar 5-EU signal to a degree consistent with acute treatment with BMH-21, a potent small-molecule inhibitor of RNAP1 [27,50] (figure 1*b,c*, compare siNT to siNT + BMH and siPOLR1A). However, it is clear that residual nucleoplasmic 5-EU signal remains even after RNAP1 inhibition (figure 1*b*, siNT + BMH and siPOLR1A), emphasizing the importance of only quantifying 5-EU staining within the nucleolus via FBL co-staining.

To achieve nucleolar 5-EU quantification during analysis, images of DNA and FBL staining (figure 1*c*, panels 1 and 2) were first used to segment nuclei and nucleoli by CellProfiler (figure 1*c*, panels 3 and 4), respectively. Then, the median 5-EU signal within each nucleolus was measured (figure 1*c*, panel 5), enabling aggregate quantification analysis per treatment condition across every nucleolus within each well (figure 1*c*, panel 6). Final calculation of mean signals, percentage inhibitions (by normalization to the negative and positive controls), and screening statistics including signal-to-background (S/B) and Z’ factor can be carried out in any standard data analysis software that can import the CellProfiler output CSV files, such as Microsoft Excel, JMP, R or Python pandas.

### 2.2. Optimization of the 5-EU assay for a miniaturized 384-well plate format

**Figure 2.**
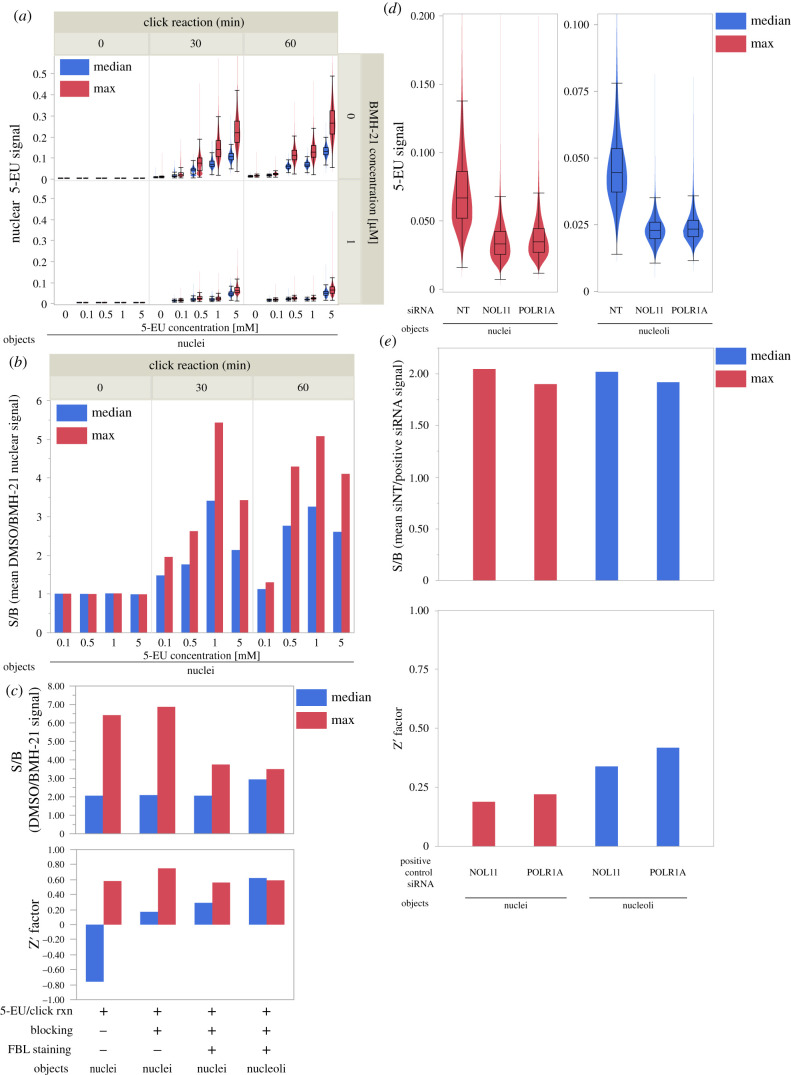
Optimization of the miniaturized 5-EU assay for nucleolar rRNA biogenesis. (*a*) Median (blue) or maximum (red) nuclear 5-EU signal for cells treated across a range of 5-EU concentrations and click reaction times, without or with BMH-21 treatment at 1 µM. *n* ≥ 20 000 cells per condition. (*b*) Control signal-to-background (S/B) ratios for treatment conditions in (*a*). Control S/B is calculated as the ratio of mean DMSO-treated nuclear 5-EU signal divided by mean BMH-21-treated nuclear 5-EU signal, for each combination of 5-EU concentration and click reaction time. Median nuclear 5-EU signal (blue), maximum nuclear 5-EU signal (red). (*c*) Control S/B and Z’ factor values for nuclei or nucleoli objects with only 5-EU visualization, 5-EU plus blocking with 10% (v/v) FBS/PBS, or 5-EU plus blocking and FBL co-staining. Median 5-EU signal (blue), maximum 5-EU signal (red). (*d*) Maximum nuclear 5-EU signal (red) or median nucleolar 5-EU signal (blue) for cells treated with siNT, siNOL11 or siPOLR1A. *n* ≥ 130 000 cells per siRNA. (*e*) Control S/B and Z’ factor values for cells (from (*d*)) treated with siNOL11 or siPOLR1A as the positive control. Control S/B is calculated as the ratio of mean siNT-treated 5-EU signal divided by mean siNOL11- or siPOLR1A-treated 5-EU signal. Maximum nuclear 5-EU signal (red), median nucleolar 5-EU signal (blue).

To adapt the 5-EU assay for use in high-throughput, we developed and optimized an optional 5-EU module that integrates into our existing nucleolar number screening platform [25]. We first investigated the assay in MCF10A cells in the absence of siRNA knockdown or FBL co-staining, using the potent RNAP1 inhibitor BMH-21 or DMSO vehicle as positive or negative controls, respectively. For the first optimization experiments without FBL co-staining, median or maximum nuclear 5-EU signal was measured. We hypothesized that maximum nuclear 5-EU signal should track nucleolar function more accurately than the median, since a larger difference in the maximum value should be observed after RNAP1 inhibition. However, both metrics should decrease significantly upon BMH-21 treatment. Based on the original 5-EU method publication [51], we chose to label cells with 5-EU for 1 h, striking a balance between signal levels and incorporation time. By varying the 5-EU treatment concentration and click reaction time in wells treated without or with BMH-21, we discovered that treatment with 1 mM 5-EU for 1 h, followed by a 30 min click reaction was optimal (figure 2*a*). Specifically, these conditions achieved the highest S/B ratio for the controls for each metric (figure 2*b*).

Next, we introduced steps to enable nucleolar segmentation including blocking with a 10% (volume-per-volume, or v/v) FBS/PBS solution and immunofluorescent staining for FBL. We used blocking and staining parameters that were previously optimized for our original screening platform [25]. Using BMH-21, we found the highest control S/B and Z′ factor occurred when measuring maximum 5-EU signal in nuclei that had been blocked but not stained for FBL (figure 2*c*, second group). To specifically quantify nucleolar 5-EU incorporation, we also measured median nucleolar 5-EU signal. We selected the median metric because, compared to the maximum, it is more robust to outliers that may occur from staining artefacts or other abnormalities. When segmenting nucleoli, a comparable Z′ factor was achieved when measuring median 5-EU signal in nucleoli (figure 2*c*, fourth group).

Interestingly, we also noted that acute treatment with 1 µM BMH-21 during our 1 h 15 min treatment period caused increased nucleoplasmic FBL staining, presumably from FBL dispersion following RNAP1 inhibition (figure 1*b*, siNT + BMH, FBL panel). We hypothesized that there may be a more optimal BMH-21 concentration where nucleolar FBL localization remains intact, but nucleolar rRNA biogenesis is still significantly inhibited.

To pursue the effects of acute BMH-21 treatment on 5-EU incorporation and FBL dispersion in more detail, we performed dose–response experiments with our optimized protocol (electronic supplementary material, figure S1). We used fourteen BMH-21 concentrations ranging from 2 nM to 20 µM to ensure sufficient capture of response dynamics. We probed BMH-21's ability to inhibit nucleolar rRNA biogenesis by quantifying both median nucleolar 5-EU signal and maximum nuclear 5-EU signal (electronic supplementary material, figure S1A,B). For these measurement schemes, we discovered an IC_50_ value of 300 ± 30 nM or 350 ± 30 nM, respectively, following BMH-21 action upon nucleolar rRNA biogenesis (electronic supplementary material, figure S1B and table S3).

We next investigated the extent to which BMH-21 decreases nucleolar-specific FBL localization (electronic supplementary material, figure S1C,D). To quantify FBL dispersion, we calculated the ratio of total area segmented as nucleoli (using FBL staining) to total area segmented as nucleus (using Hoechst staining), on a per-nucleus basis. In other words, this nucleolar/nuclear area ratio represents the percentage of each nucleus that is segmented as nucleolar by CellProfiler. We hypothesized that, as BMH-21 concentration increases and FBL disperses into the nucleoplasm, the nucleolar/nuclear area ratio would increase relative to vehicle or low concentration treatment conditions. Consistent with our hypothesis, we observed an increase in the nucleolar/nuclear area ratio from approximately 23% at low BMH-21 concentrations to approximately 41% at high BMH-21 concentrations, with an EC_50_ value of 320 ± 20 nM (electronic supplementary material, figure S1D and table S3). We find that in response to increasing BMH-21 concentration, nucleolar rRNA biogenesis is inhibited in concert with FBL dispersion, and that BMH-21's potency in both processes is approximately equivalent (electronic supplementary material, figure S1B,D,E and table S3). Thus, in our system, there is not a concentration of BMH-21 where nucleolar rRNA biogenesis is strongly inhibited that retains normal nucleolar localization of FBL. This is consistent with reports of RNAP1 inhibition resulting in nucleolar disintegration, including FBL dispersion, following acute BMH-21 treatment [27].

During our dose–response experiments, we also investigated how DMSO treatment affects nucleolar rRNA biogenesis and FBL localization. Importantly, we find that treatment with 1 µl of DMSO vehicle (approximately 2% [v/v]) decreases median nucleolar 5-EU signal and maximum nuclear 5-EU signal by 10–15% (electronic supplementary material, figure S1F). This inhibitory effect of DMSO is not unexpected, as low-dose DMSO treatment has been shown to alter RNA structure *in vitro* [52] and to reduce viability and induce apoptosis after 24 h *in vivo* [53]. Following DMSO treatment, we did not notice an effect on FBL localization as reported by nucleolar/nuclear area ratio (electronic supplementary material, figure S1G).

We caution that the accuracy of nucleolar segmentation should be closely monitored by calculating nucleolar/nuclear area ratio, if using BMH-21 or another potent RNAP1 inhibitor that causes FBL dispersion. Aberrancies in FBL staining could lead to inaccurate segmentation, affecting results obtained by calculating median nucleolar 5-EU signal. In these situations, maximum nuclear 5-EU signal can be monitored in addition to or in place of median nucleolar 5-EU signal. If using the assay to study the effects of DMSO-solubilized small molecules, care should also be taken to treat all wells with equal volumes of vehicle, as DMSO treatment does slightly affect 5-EU incorporation.

In the final phase of optimization, we studied how siRNA knockdown of known RB factors affected nuclear and nucleolar 5-EU signal. We chose to deplete NOL11, a small subunit processome factor critical for pre-rRNA transcription [54], or POLR1A, the largest subunit of the RNAP1 complex, as positive controls. We verified robust knockdown of *NOL11* or *POLR1A* mRNA transcripts using RT-qPCR (electronic supplementary material, figure S2). Compared to treatment with siNT, the depletion of NOL11 or POLR1A decreased maximum nuclear signal and median nucleolar signal by roughly 50% in each case (figure 2*d*), corresponding to control S/B values of 1.9–2.0 for each control (figure 2*e*, top). However, measuring median nucleolar signal had lower object-to-object variability, resulting in more favourable Z’ factors than when measuring maximum nuclear signal (figure 2*e*, bottom). Thus, both NOL11 and POLR1A are excellent positive controls for inhibiting nucleolar rRNA biogenesis in the 5-EU assay, when median nucleolar signal is measured. In follow-up validation studies (see below), we confirmed that measuring the median nucleolar 5-EU signal provides the most robust Z′ factors, despite the nucleolar 5-EU standard deviation metric having a higher control S/B ratio (electronic supplementary material, figure S3). From these results, we recommend measuring maximum nuclear 5-EU when using treatments that cause FBL dispersion, such as BMH-21. We also conclude that measuring median nucleolar 5-EU signal, which corresponds only to nucleolar rRNA biogenesis, is the optimal 5-EU assay endpoint under conditions where FBL has sufficiently specific nucleolar localization, as optimized by the assay user for a given combination of experimental variables including cell line and treatment conditions.

### 2.3. Validation of the high-throughput 5-EU assay on 68 known ribosome biogenesis factors

**Figure 3.**
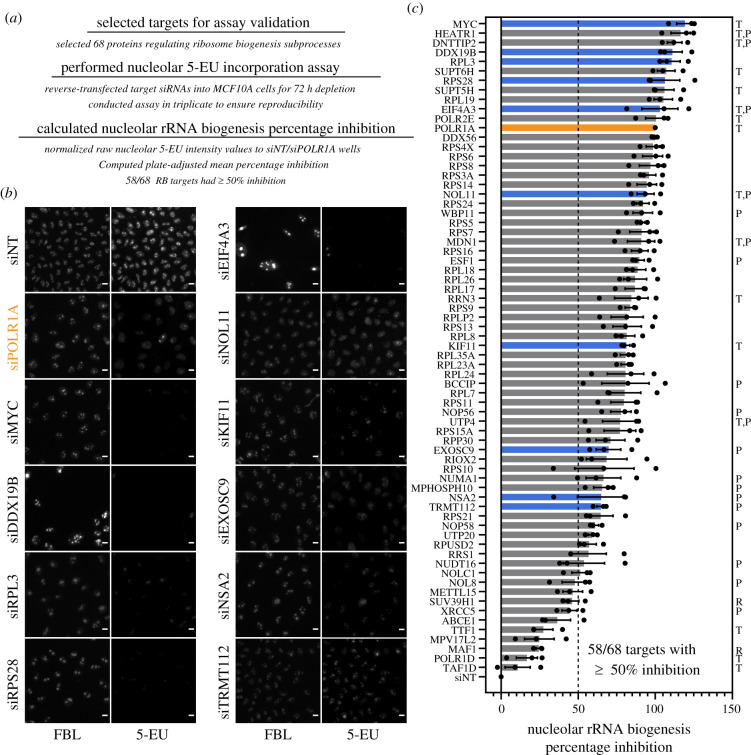
Validation of the 5-EU assay for nucleolar rRNA biogenesis on 68 known RB factors. (*a*). Outline of assay validation experiments. Sixty-eight proteins known to regulate RB subprocesses, including RNAP1 transcription and pre-rRNA processing, modification or stability, were selected for assay validation. The 5-EU assay was performed on cells depleted of these factors in biological triplicate, as described. (*b*). Representative images of FBL staining and 5-EU visualization for cells treated with siNT (negative control), siPOLR1A (positive control, orange), or a subset of siRNAs targeting known RB factors. (*c*) Nucleolar rRNA biogenesis percentage inhibition values for cells depleted of each known RB factor. Black dots, individual percentage inhibition values for one biological replicate. Solid bars, mean percentage inhibition (*n* = 3). Orange bar, POLR1A positive control (percentage inhibition = 100%). Blue bars, RB factors illustrated in (*b*). Letters to right indicate factors involved in RNAP1 transcription (T), pre-rRNA processing (P), or transcription repression (R).

After optimization, we validated the high-throughput 5-EU assay using a subset of 68 previously studied RB factors, including RPs and assembly factors for both ribosomal subunits, as well as core RNAP1 machinery and drivers of transcription such as MYC (figure 3*a*; electronic supplementary material, table S1). We depleted each RB factor over 72 h using siRNA pools in accordance with our protocol, performing the assay in biological triplicate to ensure reproducibility. Strikingly, we found that depletion of 58/68 biogenesis factors led to a significant (greater than or equal to 50%) inhibition of nucleolar 5-EU signal after standardization to the controls (figure 3*a*). Images of the assay controls illustrate typical signal levels observed for the negative control siNT, set at 0% inhibition, and the positive control siPOLR1A, set at 100% inhibition (figure 3*b*, siNT and siPOLR1A). Furthermore, images from the RB factors tested demonstrate the sensitivity of the assay to RNAP1 inhibition, from extreme effects above 100% inhibition (e.g. siMYC) to more moderate inhibitory effects (e.g. siTRMT112) (figure 3*b*). Full results from the assay validation are presented in figure 3*c* and electronic supplementary material, table S1.

Strikingly, we observed 11 targets that resulted in stronger nucleolar rRNA biogenesis inhibition than the positive control, POLR1A; consistent with a mean percentage inhibition greater than 100%, 7/11 of these targets are implicated in control of pre-rRNA transcription, including MYC [55], HEATR1/UTP10 [56–58], DNTTIP2/TdIF2 [59], SUPT6H [60], SUPT5H [25], EIF4A3/DDX48 [61] and POLR2E [62]. Overall, 12/58 factors with a significant inhibition of nucleolar rRNA biogenesis have been implicated in transcription, also including the RNAP1 initiation factor RRN3 [63–65], two other t-UTPs, NOL11/UTP8 [54] and UTP4 [66], and the proteins MDN1, a pre-60S assembly factor, and KIF11, a mitotic kinesin essential for RB [24].

Pre-rRNA processing and modification factors comprised a sizeable subset of factors with significant nucleolar rRNA biogenesis mean percentage inhibition. In total, 19/58 factors that inhibited nucleolar rRNA biogenesis were critical for processing, including the t-UTPs HEATR1/UTP10, NOL11/UTP8 and UTP4 [54,56–58,66], the C/D box snoRNP scaffolds NOP56 and NOP58 [67], as well as other processing factors including DNTTIP2 [23], WBP11 [68], MDN1 [69], ESF1 [23,70], BCCIP [71], RPP30 [23,72], EXOSC9 [73], NUMA1 [25], MPHOSPH10 [74], TRMT112 [75], UTP20 [76] and NUDT16 [77]. In addition, nucleolar rRNA biogenesis was moderately inhibited for the pre-rRNA modification factors TRMT112 [75], RPUSD2 [68] and NOLC1 [78,79]. Notably, factors involved in transcription had a higher mean percentage inhibition than factors involved in processing (83.1% inhibition versus 74.9% inhibition, *n* = 15 versus *n* = 22); factors involved in both transcription and processing had a mean percentage inhibition of 99.0% (*n* = 6).

We also noted significant percentage inhibition averages for 28 RPs from both the 40S and 60S subunits. Almost all RPs are essential for pre-rRNA biogenesis in the yeast *Saccharomyces cerevisiae* [80,81] and in human cells [12,82], compatible with a concomitant observed decrease in nucleolar rRNA biogenesis following their depletion.

Furthermore, of the 10 factors that had a mean percentage inhibition value under 50%, five factors were either inhibitors of pre-rRNA transcription, including SUV39H1 [83] and MAF1 [84,85], mitochondrial RB factors, including METTL15 [86,87] and MPV17L2 [88], or ribosome recycling factors involved in translation, namely ABCE1 [89].

Surprisingly, the other five RB factors with a mean percentage inhibition less than 50% are well-appreciated for playing roles in pre-rRNA transcription, including POLR1D [90], TAF1D [91] and TTF1 [92], and in pre-rRNA processing, including NOL8 [93] and XRCC5/Ku86, which also aids TTF1 during RNAP1 termination [94,95]. It is possible that these factors were not significantly depleted following transfection, or that, within our timeframe, the 5-EU assay cannot detect a significant change in nucleolar RNA levels as a result of non-concordant changes in both pre-rRNA transcription and stability.

## 3. Discussion

More precise, accessible methods for the study of nucleolar function are critical for illuminating novel RB regulators and next-generation therapeutics for human disease states including cancer, ageing, and rare ribosomopathies. Here, we developed an HT-ready, image-based assay that selectively measures nucleolar rRNA biogenesis in MCF10A breast epithelial cells. Building upon previous HTS techniques, we combined FBL staining of nucleoli and 5-EU incorporation into nascent RNA to measure only the 5-EU signal corresponding to nucleoli. We optimized the parameters of this assay using both small-molecule inhibition (BMH-21) and acute siRNA depletion of the essential RNAP1 transcription machinery (POLR1A and NOL11). Our detailed assay framework can be applied to studies of novel RNAP1 drug inhibitors and cellular regulators of nucleolar rRNA biogenesis, with the potential for adaptation to a variety of cell types. Our assay will increase the dimensionality and efficiency of future HTS campaigns focused on the nucleolus, accelerating the discovery of novel modulators of nucleolar function,

After optimizing the 5-EU assay for a miniaturized format, we validated its utility on 68 known RB factors including core RNAP1 components, small (pre-40S) or large (pre-60S) ribosomal subunit-specific processing and assembly factors, pre-rRNA modification factors and RPs. As expected, all RB factors had a percentage inhibition value greater than 0%. While a wide range of percentage inhibition values was observed, 58/68 factors (85.5%) had a mean percentage inhibition of at least 50%, signalling that the 5-EU assay robustly reports depletion conditions that interrupt nucleolar rRNA biogenesis.

Although our nucleolar 5-EU assay accurately reported the interruption of nucleolar rRNA biogenesis for the vast majority of RB factors studied, we note the following considerations and caveats regarding our method and results. First, nucleolar rRNA biogenesis can be affected by changes in one or more RB subprocesses including pre-rRNA transcription, processing, modification, and binding by RPs, which all occur co-geographically within the nucleolus. Since kinetic studies have defined the rates of human pre-rRNA transcription [96] and initial pre-rRNA processing steps [97] to be on the order of minutes, 5-EU label will be distributed across a population of partially processed or folded nucleolar pre-rRNA intermediates at the end of the assay's 1 h labelling period. Therefore, nucleolar 5-EU incorporation over the course of 1 h cannot report solely on RNAP1 transcriptional activity, and additional mechanistic assays may be necessary to precisely define how an experimental treatment alters RB following the observation of a 5-EU defect. We highlight the importance of our discovery of the expanded ability of the 5-EU assay to report on defects in multiple RB steps in addition to RNAP1 transcription, which to our knowledge has not been previously considered. Second, a treatment, like 72 h siRNA-mediated depletion of cultured human cells as we have done here, may also have opposing, compensatory effects on multiple RB subprocesses, leading to an artificially low percentage inhibition and a false-negative result. More broadly, as with any HTS study using RNAi-mediated target depletion, off-target effects or inefficient on-target depletion could lead to false positive or false-negative results, respectively [98,99]. Second, we have empirically defined a percentage inhibition significance cutoff of 50% inhibition because it minimizes the number of incorrectly classified RB factors. However, it is still unclear if there is a more stringent percentage inhibition cutoff that would correspond strictly to RB factors regulating RNAP1 transcriptional activity, or cutoffs for other RB subprocesses. Future studies may elucidate the relationship between the roles of a given RB factor and the nucleolar rRNA biogenesis percentage inhibition value observed upon its depletion. Finally, close attention must be paid to the accuracy of nucleolar segmentation if median nucleolar 5-EU signal is being quantified; the maximum nuclear 5-EU signal metric can be used if treatment causes significant FBL dispersion, as we have observed with BMH-21 at 1 µM.

Our miniaturized 5-EU assay enables direct quantification of nucleolar rRNA biogenesis in high-throughput, providing clearer insight into how targets modulate RB and improving upon previous HTS techniques for studying nucleolar function. The 5-EU assay is also compatible with our previously published assay for a nucleolar number [25] and is likely to be compatible with other high-content assays for RB that monitor nucleolar architecture by co-staining for nucleolar proteins [32,33]. By extending the dimensionality and specificity of current state-of-the-art assays which indirectly track nucleolar function, our 5-EU assay will permit researchers to focus on the most promising screen candidates earlier, thereby increasing the efficiency of RB-directed screening campaigns. We anticipate that the miniaturized 5-EU assay will expedite the identification and definition of novel regulators of RB in basic or translational studies of nucleolar function.

## 4. Material and methods

### 4.1. Cell lines and culture conditions

Human MCF10A breast epithelial cells (ATCC CRL-10317) were cultured in DMEM/F-12 (Gibco 11330032) with 5% horse serum (Gibco 16050122), 10 µg ml^−1^ insulin (MilliporeSigma I1882), 0.5 µg ml^−1^ hydrocortisone (MilliporeSigma H0135), 20 ng ml^−1^ epidermal growth factor (Peprotech AF-100-15), and 100 ng ml^−1^ cholera toxin (MilliporeSigma C8052). Cells were incubated at 37°C in a humidified atmosphere with 5% CO_2_.

### 4.2. RNAi depletion by reverse-transfection

RNAi depletion was conducted in MCF10A cells as previously reported [24,25]. MCF10A cells were reverse-transfected into an arrayed 384-well plate library containing small interfering RNA (siRNA) constructs (Horizon Discovery, see electronic supplementary material, table S2). Assay-ready plates containing 10 µl of 100 nM ON-TARGET siRNAs resuspended in 1X siRNA buffer (Horizon Discovery B-002000-UB-100) were prepared from master library 384-well plates (Horizon Discovery, 0.1 nmol scale) and stored at −80°C. Plates were thawed at room temperature for 30 min and briefly centrifuged at 300 RPM. siRNA controls (electronic supplementary material, table S2) were freshly diluted in 1 X siRNA buffer to 100 nM from a 50 µM frozen stock, and 10 µl of 100 nM control siRNAs were manually pipetted into the assay-ready plates. To each well, 10 µl of a 1 : 100 (v/v) RNAiMAX:OptiMEM solution was added (Invitrogen 13778-150, Gibco 31985070), after which the plates were briefly centrifuged at 300 RPM and incubated at room temperature for 30 min. MCF10A cells at 70%-80% confluency were trypsinized for 15 min with 0.05% trypsin (Gibco 25300054), resuspended in culture media, counted with a hemacytometer, and diluted in culture medium to a density of 100 000 cells ml^−1^. Thirty microliters of cells were dispensed into assay plates using a Multidrop Combi Reagent Dispenser (Thermo Scientific), to achieve a seeding density of 3000 cells well^−1^, a final volume of 50 µl and a final siRNA concentration of 20 nM. Seeded assay plates were briefly centrifuged at 300 RPM and incubated at 37°C for 72 h. RB factors were screened in triplicate.

### 4.3. Analysis of mRNA knockdown by RT-qPCR

MCF10A cells were seeded at 1 × 10^5^ cells per well in 6-well plates and incubated at 37°C for 24 h. Cells were reverse transfected with 20 nM siRNA controls (electronic supplementary material, table S2) using lipofectamine RNAiMAX per manufacturer's instructions for 72 h. RNA was harvested using TRIzol reagent (Life Technologies 15596018) per manufacturer's instructions. RNA used for cDNA synthesis had a minimum A_260_/A_230_ ratio of 1.7. cDNA was synthesized from 1 µg total input RNA using iScript gDNA Clear cDNA Synthesis Kit (BioRad 1725035). qPCR was performed using SYBR Green reagent (BioRad 1725121) and gene-specific primers (shown below). Cycling parameters were as follows: initial denaturation 95°C for 30 s, 40 cycles 95°C for 15 s and 60°C for 30 s, melt curve analysis 60°C to 94.8°C in 0.3°C increments. Data analysis was completed using the comparative C_T_ method (*ΔΔ*C_T_) using *ACTB* mRNA as an internal control.

**Table:** 

target gene	forward primer sequence	reverse primer sequence
	(5′ → 3′)	(5′ → 3′)
*ACTB*	ATT GGC AAT GAG CGG TTC	CGT GGA TGC CAC AGG ACT
*NOL11*	TCC AGG CAA GAA CGG TGT TT	GAA ACC TGC AGT CCT ACC CC
*POLR1A*	CTT CAT TCT TCC ACA GGG CA	CCG AAA GGA ACA CAA CAG CA

### 4.4. BMH-21 treatment and 5-ethynyl uridine incorporation

BMH-21 (MilliporeSigma SML1183) was resuspended in DMSO to a working concentration of 50 µM (50X) and stored at −20°C. 5-EU (ClickChemistryTools 1261-100) was resuspended in ddH_2_O from powder to a working concentration of 50 mM (50X) and stored at −20°C. For BMH-21 treatment, reverse-transfected assay plates were treated 15 min before the end of the 72 h RNAi depletion period. One microliter of either DMSO vehicle or of 50 µM BMH-21 was manually added directly to 50 µl medium in the appropriate wells of the assay plates, which were then briefly centrifuged at 300 RPM and incubated for 15 min before 5-EU incorporation and for the remaining 1 h 5-EU treatment period. For 5-EU incorporation into nascent RNA, reverse-transfected assay plates were treated for 1 h after the end of the 72 h RNAi depletion period. One microlitre of 50 mM 5-EU was manually added directly to 50 µl medium in each well of the assay plates, which were then briefly centrifuged at 300 RPM and incubated for 1 h.

### 4.5. Immunofluorescent staining and click fluorophore labelling

After 5-EU incorporation, cells were gently washed with 30 µl of PBS and fixed with 1% (v/v) paraformaldehyde (Electron Microscopy Sciences 15710-S) diluted in PBS at room temperature for 20 min. Cells were washed twice with 20 µl wash buffer consisting of PBS with 0.05% (v/v) TWEEN 20 (MilliporeSigma P1379), then permeabilized with 20 µl of 0.5% (v/v) Triton X-100 in PBS for 5 min. Cells were washed twice with 20 µl wash buffer and incubated with 20 µl of blocking buffer consisting of 10% (v/v) FBS (MilliporeSigma F0926) diluted in PBS for 1 h at room temperature. FBL primary antibody solution was prepared by diluting supernatant from the 72B9 hybridoma line [100] at 1 : 500 or 1 : 250 (v/v) in blocking buffer. After blocking, cells were incubated with 20 µl FBL primary antibody solution for 2 h at room temperature. Cells were washed twice with 20 µl wash buffer and incubated with 20 µl secondary antibody solution, consisting of 1 : 1000 (v/v) goat anti-mouse AlexaFluor 647 (Invitrogen A-21236) and 3 µg ml^−1^ Hoechst 33342 dye in blocking buffer, for 1 h in the dark at room temperature. Immediately before the end of the secondary antibody incubation period, the click reaction cocktail was prepared in PBS by combining 5 µM AFDye 488 azide (ClickChemistryTools 1275-5), 0.5 mg ml^−1^ CuSO_4_ (Acros Organics 197730010) and 20 mg ml^−1^ freshly resuspended sodium ascorbate (Alfa Aesar A15613). Cells were washed twice with 20 µl wash buffer, then treated with 20 µl of click reaction cocktail for 30 min in the dark at room temperature. Cells were washed twice with 20 µl wash buffer and soaked in 20 µl PBS containing 3 µg ml^−1^ Hoechst 33342 dye for 30 min in the dark at room temperature to dissociate excess AFDye 488 azide. Cells were washed twice with 20 µl wash buffer and 40 µl of PBS was added to each well before high-content imaging.

### 4.6. High-content imaging

Stained assay plates were imaged with a GE Healthcare IN Cell Analyzer 2200. Fields of view were acquired at 20 X magnification with 2 × 2 pixel binning (665.63 µm × 665.63 µm, 1 pixel = 0.65 µm) at 16-bit depth using Cy5, DAPI, and FITC channels for FBL, Hoechst, and 5-EU staining, respectively. Laser autofocus was used to automatically determine imaging Z-height. For publication, images were cropped, merged and labelled with scale bars using ImageJ 1.53i [101].

### 4.7. Cellprofiler pipeline and data analysis

Image analysis was conducted with a custom pipeline for CellProfiler 3.1.9 [47,102] (electronic supplementary material, file S1). Briefly, nuclei and nucleoli objects were segmented from DAPI and Cy5 channels, respectively, using global two-class Otsu thresholding. Child nucleoli objects were linked to parent nuclei objects using the RelateObjects module. For both object classes, area was measured from DAPI or Cy5 images, and 5-EU intensity was measured from FITC images. Object-level normalized 5-EU intensity metrics including maximum, mean, median, and standard deviation were calculated by CellProfiler. Raw CellProfiler output CSV files including plate metadata were imported into and analysed with JMP Pro 15.2.0 (SAS Institute). Per-well averages were computed for each 5-EU metric. For each plate, aggregate control well data were used to calculate signal-to-background (S/B) and Z’ factor screening statistics. Nucleolar rRNA biogenesis percentage inhibition values were calculated for each well as follows:

$$\mathrm{nucleolar}\;\mathrm{rRNA}\;\mathrm{biogenesis}\;\mathrm{percentage}\;\mathrm{inhibition}=\left(1-\frac{x_{i}-{\bar{x}}_{POLR1A}}{{\bar{x}}_{NT}-{\bar{x}}_{POLR1A}}\right)\times 100\text{\%},$$

where *x* is the average 5-EU metric value over all objects in a well, $x_{i}$ is the well metric value for a non-control well, ${\bar{x}}_{NT}$ and ${\bar{x}}_{POLR1A}$ are averages of all NT or POLR1A control well metric values, respectively. Plate-adjusted percentage inhibition values were calculated for non-control wells by subtracting the plate's median NT percentage inhibition value from each non-control well percentage inhibition [103]. Nucleolar/nuclear area ratios were calculated for each nucleus by summing the area of all child nucleoli for a given nucleus, then dividing by the area of the specified nucleus. Nucleoli without a valid parent nucleus (parent ID 0) were discarded. Per-well averages were then computed. Optimization data were graphed in JMP. Triplicate data from the RB factor screen were averaged in JMP and graphed with GraphPad Prism 8 (GraphPad Software).

### 4.8. BMH-21 dose–response treatment

A 14-point 50X dilution series ranging from 1 mM to 100 nM BMH-21 was created in DMSO vehicle from a 1 mM BMH-21 working stock. In a 384-well plate, 3000 MCF10A cells per well were plated in 50 µl of media on day 0. On day 1, columns 3–4 were treated with 1 µl of only vehicle and each column from 5 to 18 was treated with 1 µl of one concentration of the BMH-21 dilution series for 15 min at 37°C, resulting in a 1X dilution series ranging from 20 µM to 2 nM at final concentration. Columns 1–2 were not treated with DMSO. Each well was treated with 1 µl of 50 mM 5-EU for a final concentration of 1 mM 5-EU for an additional 1 h at 37°C. Cells were fixed and stained as detailed above. Rows A–H were stained only for FBL (no 5-EU click reaction), and rows I-P were stained for FBL and treated with the 5-EU click reaction. Cells were imaged as above and processed with the CellProfiler pipeline. Raw data were analysed in JMP, and per-well averages were used to fit dose–response curves using JMP's Logistic 4 Parameter Hill equation. Fit parameter estimates and errors are provided in the electronic supplementary material, table S3. Summary data were graphed in JMP.

## Data accessibility

5-EU data, siRNAs used, and dose–response fit data are included in the electronic supplementary material, tables S1, S2 and S3, respectively. Our CellProfiler computational pipeline is also included as Supplementary File 1, and an optimized 5-EU assay standard operating procedure is included as Supplementary File 2.

The data are provided in the electronic supplementary material [104].
